# Physical Activity and Inhibitory Control: The Mediating Role of Sleep Quality and Sleep Efficiency

**DOI:** 10.3390/brainsci11050664

**Published:** 2021-05-19

**Authors:** Lin Li, Qian Yu, Wenrui Zhao, Fabian Herold, Boris Cheval, Zhaowei Kong, Jinming Li, Notger Mueller, Arthur F. Kramer, Jie Cui, Huawei Pan, Zhuxuan Zhan, Minqiang Hui, Liye Zou

**Affiliations:** 1 Key Laboratory Ministry of Education of Adolescent Health Assessment and Exercise Intervention, East China Normal University, Shanghai 200241, China; lilin.xtt@163.com (L.L.); zhaowenrui108@163.com (W.Z.); cuijie1615@163.com (J.C.); 13758114178@163.com (H.P.); zzx951105@163.com (Z.Z.); m13213595838@163.com (M.H.); 2 School of Physical Education and Health, East China Normal University, Shanghai 200241, China; 3 Institute of KEEP Collaborative Innovation, Shenzhen University, Shenzhen 518060, China; yuqianmiss@163.com (Q.Y.); jinmingli1999@gmail.com (J.L.); 4 Exercise Psychophysiology Laboratory, School of Psychology, Shenzhen University, Shenzhen 518060, China; 5 Department of Neurology, Medical Faculty, Otto von Guericke University, Leipziger Str. 44, 39120 Magdeburg, Germany; fabian.herold@st.ovgu.de (F.H.); notger.mueller@dzne.de (N.M.); 6 Research Group Neuroprotection, German Center for Neurodegenerative Diseases (DZNE), Leipziger Str. 44, 39120 Magdeburg, Germany; 7 Swiss Center for Affective Sciences, University of Geneva, 1205 Geneva, Switzerland; Boris.Cheval@unige.ch; 8 Laboratory for the Study of Emotion Elicitation and Expression (E3Lab), Department of Psychology, FPSE, University of Geneva, 1205 Geneva, Switzerland; 9 Faculty of Education, University of Macau, Macao 999078, China; zwkong@um.edu.mo; 10 Center for Cognitive and Brain Health, Department of Psychology, Northeastern University, Boston, MA 02115, USA; a.kramer@northeastern.edu; 11 Beckman Institute, University of Illinois at Urbana-Champaign, Champaign, IL 61801, USA

**Keywords:** physical activity, sleep, inhibitory performance, mediating effects

## Abstract

Objectives: the current study aimed to investigate the relationship between physical activity (PA) level and inhibitory control performance and then to determine whether this association was mediated by multiple sleep parameters (i.e., subjective sleep quality, sleep duration, sleep efficiency, and sleep disturbance). Methods: 180 healthy university students (age: 20.15 ± 1.92 years) from the East China Normal University were recruited for the present study. PA level, sleep parameters, and inhibitory control performance were assessed using the International Physical Activity Questionnaire (IPAQ), the Pittsburgh Sleep Quality Index Scale (PSQI), and a Stroop test, respectively. The data were analyzed using structural equation modeling. Results: A higher level of PA was linked to better cognitive performance. Furthermore, higher subjective sleep quality and sleep efficiency were associated with better inhibitory control performance. The mediation analysis revealed that subjective sleep quality and sleep efficiency mediated the relationship between PA level and inhibitory control performance. Conclusion: our results are in accordance with the literature and buttress the idea that a healthy lifestyle that involves a relatively high level of regular PA and adequate sleep patterns is beneficial for cognition (e.g., inhibitory control performance). Furthermore, our study adds to the literature that sleep quality and sleep efficiency mediates the relationship between PA and inhibitory control performance, expanding our knowledge in the field of exercise cognition.

## 1. Introduction

Brain health and cognitive enhancement are important factors for successful studies, better career opportunities, and a higher quality of life and thus has received widespread attention from different disciplines including medicine and education [1]. Emerging evidence demonstrates that regular physical activity (PA) can enhance cognitive performance, especially in the domain of executive functioning (EF) [2,3,4,5]. Among the diverse subcomponents of EF, controlled inhibition (the capacity to suppress irrelevant information or prepotent responses in monitoring and updating information) has been reported to benefit more from PA than other aspects of EF (e.g., shifting or updating) [6]. Considering that the brain develops until the age of 30 years [7,8,9,10] and the important role of inhibitory control in mastering life successfully [11], there is an urgency to investigate how regular PA influences inhibitory control performance and how to maximize PA-induced cognitive benefits among youths. Of note, existing research mostly focuses on children and older individuals, while there is a lack of studies on young to middle-aged adults [1]. Additionally, although PA has been proven to maintain and improve neurocognitive function, much less is known about the pathway by which it exerts salutary effects on inhibitory control performance. Among the suggested potential mediators, sleep has been proposed as an ideal candidate to explain the PA–cognition links, although empirical evidence in younger adults is currently lacking [12].

The improved cognitive performance among individuals who engaged in regular PA may be explained, at least partly, by sleep health. Indeed, young adults regularly experience restricted sleep (70% of people sleeping less than 6 h at least one night a week) due to educational, vocational, and social responsibilities [13]. Mounting evidence shows that the increased PA contributes to sleep health (i.e., sleep duration, efficiency, and quality). For example [14,15], randomized controlled trials conducted in 1997 were the first to indicate the PA (i.e., moderate-intensity exercise, daily activity)-induced benefits in self-reported sleep quality. A review by Vanderlinden et al. indicated that moderate intensity exercise intervention (lasted 12 weeks to 6 months), with a frequency of three times per week, showed the highest improvements in sleep outcomes in older adults [16]. Even among patients with sleep disruptions, exercise intervention was also found to positively influence sleep outcomes [17].

The provided evidence supports the idea that better sleep was linked to improved cognitive performance [18,19,20]. For example, sufficient sleep quality has been shown to be essential for an optimal cognitive performance, especially in the domain of EF (i.e., inhibitory control) [21]. However, although the exact neurobiological mechanisms of the association between sleep and cognitive performance are not fully understood. Sleep might work through complex ultradian, circadian, and homeostatic regulations to positively influence the prefrontal circuits that are known to be important neural correlates for EF [22,23,24]. The improved prefrontal circuits could be well reflected by higher voltage and slower brain waves during non-rapid eye movement (NREM), coinciding with the deactivation of dorsolateral prefrontal cortex during rapid eye movement (REM) [25].

To the best of our knowledge, there are only two studies examining the potential mediating role of sleep health on the association between PA and cognitive function [26,27]. Wilckens et al. found that sleep efficiency may be one pathway by which PA benefits executive control, but this study only investigated two sleep health indicators (sleep efficiency and total sleep time as measured by accelerometer-based sleep assessment) [26]. Contrary to the outcomes of Wilckens et al., Falck et al. reported that PA and sleep quality interacted with cognitive function via independent mechanisms without mediation effects [27] among the elderly (above 55 years). To address the gap and inconsistency, the current study attempts to explore how PA influences young adults’ inhibitory control via a wider range of sleep parameters [28]. We aimed (i) to investigate the relationships among regular PA level, inhibitory control and multiple sleep parameters (subjective sleep quality, sleep duration, sleep efficiency, and sleep disturbance) among university students and (ii) to examine how sleep parameters mediate the relationship between PA level and inhibitory control performance.

## 2. Methods

### 2.1. Participants

In total, 180 healthy university students (age: 20.15 ± 1.92 years; 109 females and 71 males) from the East China Normal University were recruited in the present study. The PA level of participants, their sleep status, and their inhibitory control performance were assessed via the International Physical Activity Questionnaire (IPAQ), the Pittsburgh Sleep Quality Index Scale (PSQI), and Stroop test, respectively. Moreover, all participants were screened for depression and anxiety on the Self-rating depression scale (SDS) and the Self-Rating Anxiety Scale (SAS). Exclusion criteria included (1) SDS score ≥ 53, (2) SAS score ≥ 50, (3) failure to complete all of the assessments, and (4) left-handedness. The whole procedure was approved by the ethic committee of the East China Normal University (No. HR 085-2018), and all participants signed informed consent before the onset of this experiment.

### 2.2. Demographic Information

A self-developed scale on demographic information was used to acquire participants’ information such as gender, age, height, and weight. Height and weight were assessed by a Height Weight Meter. Body Mass Index (BMI) was calculated as weight (in kilograms) divided by the square of height (in meters).

### 2.3. International Physical Activity Questionnaire

The IPAQ was used to assess the PA level of each individual (content validity = 0.98, calibration validity = 0.41, and test–retest reliability = 0.78). The IPAQ totally consists of five parts: (1) job-related PA; (2) transportation PA; (3) housework, house maintenance, and caring for family; (4) recreation, sport, and leisure-time PA; and (5) time spent sitting. MET (Metabolic Equivalent of Task) minutes per week, as a continuous variable, represents the amount of energy expended carrying out physical activity.

### 2.4. Pittsburgh Sleep Quality Index Scale

The subjective level of sleep quality was assessed using the PSQI, which queries possible factors related to sleep disturbance over the past four weeks [29]. It includes 19 items within seven sleep-related domains: subjective sleep quality, sleep latency, sleep duration, habitual sleep efficiency, sleep disturbances, use of sleep medication, and daytime dysfunction [29]. The score of each domain is rated from 0 to 3 and seven sub-scores can be added to produce a total sleep quality score ranging from 0 to 21 [29], with higher total score indicating lower sleep quality [29].

### 2.5. Measurement of Inhibitory Control Performance

**Figure 1:**
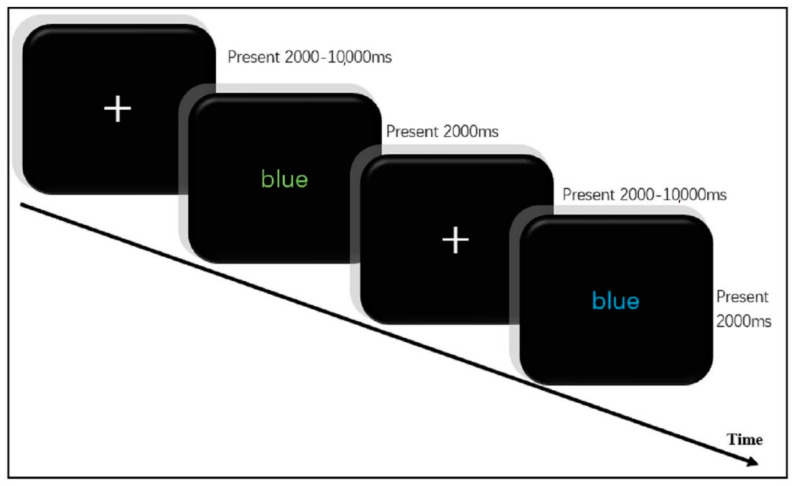
Stroop test.

Inhibitory control performance was tested by a Stroop task (Figure 1) in which participants were asked to differentiate stimulation in the mixed congruent and incongruent conditions [30]. The stimuli used in this task were composed of four words (red, yellow, blue, and green in Chinese). In the congruent condition, the word was shown in matching colors; in the incongruent condition, the word did not match the color. The task lasted for 240 s, including two blocks with 32 trials (8 congruent and 24 incongruent trails) in total. In each trial, a fixed cross was presented first to attract participants’ attention and then the color words were shown randomly, with each one lasting for 2000 ms. The inter-stimulus intervals were also random (2000, 4000, 6000, 8000, or 10,000 ms). The participants were asked to report the correct color of the word by pressing the corresponding button on a keyboard as quickly and accurately as possible, ignoring the interference by the word’s semantic meaning. Subjects performed 20 practice trials before performing the experimental trial blocks. Accuracy and reaction time of each condition were also recorded. According to the operational definition of inhibitory performance in the Stroop test, the efficiency of inhibitory performance was characterized by the difference in reaction time. The smaller the Stroop effect (ΔRT = RT_incongruent_ − RT_congruent_), the stronger the inhibitory performance.

### 2.6. Statistical Analysis

Statistical analyses were conducted using SPSS (Statistical Product and Service Solutions) software, version 23. Mean and standard deviation for all variables were calculated. Unless noted, all effects were described as significant at *p* < 0.05. Partial correlation analyses, with sex, age, and BMI as covariates, were used to examine the relationships between PA level (MET score), inhibitory control performance, and seven sleep parameters (subjective sleep quality, sleep latency, sleep duration, habitual sleep efficiency, sleep disturbances, use of sleep medication, and daytime dysfunction). Moreover, structural equation modeling (SEM) with the help of AMOS (Analysis of Moment Structure) was conducted to assess whether the sleep parameters mediated the association between PA and inhibitory control performance. The indices used to evaluate the overall fit of model included Chi-square (χ^2^) test, normed Chi-square (χ^2^/df), normed fit index (NFI), incremental fit index (IFI), and comparative fit index (CFI). Usually, a well-fitted model should meet the following requirements: (1) a nonsignificant Chi-square; (2) 0.90 or less NFI, IFI, and CFI values; and (3) 0.06 or less normed Chi-square value [31]. The paths from the mediation model were examined using direct and indirect effects of study variables (PA level, sleep parameters, and the Stroop effect); bootstrapping approach was used to test the statistical significance of mediation.

## 3. Results

The results of the demographic and anthropometric parameters, the IPAQ, the PSQI, and the performance on the Stroop test are shown in Table 1.

### 3.1. Correlation Analysis

As shown in Table 2, the results indicate negative correlations of regular PA level with (1) subjective sleep quality (*r* = −0.42, *p* < 0.001), (2) habitual sleep efficiency (*r* = −0.52, *p* < 0.001), and (3) the Stroop effect (*r* = −0.39, *p* < 0.001) (with a higher score in the PSQI indicating worse sleep status and a smaller Stroop effect indicating better inhibitory control performance). Additionally, the scores of subjective sleep quality and sleep efficiency were found to positively correlate with the Stroop effect (*r* = 0.83, *p* < 0.001; *r* = 0.61, *p* < 0.001).

### 3.2. Multiple Mediation Model

**Figure 2:**
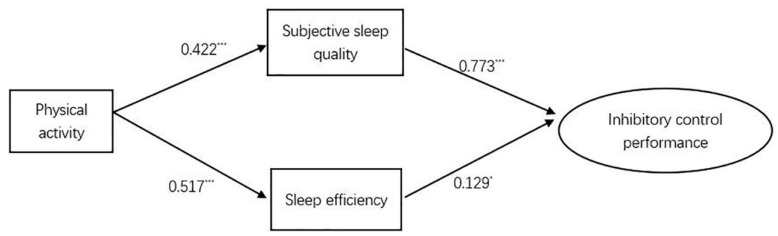
Multiple mediation model 2. Note. * means that *p* value is less than 0.05; *** means that *p* value is less than 0.001.

Based on the outcomes of correlation analyses, only two sleep parameters (subjective sleep quality and sleep efficiency) were included in the mediation model. The structured equation model was used to determine the mediating role of sleep parameters on the relationship between PA and inhibitory control (Figure 2). In the initial model, RMSEA (0.07) and SRMR (0.06) were higher than 0.05, which meant that the initial model failed to present a good fit. Therefore, the model was modified as specified by modification indices (χ^2^/df = 64.43, NFI = 0.83, IFI = 0.83, and CFI = 0.82). After removing the nonsignificant path between physical activity and inhibitory control performance, the model was re-analyzed and all indices within the new model show a good fit (χ^2^/df = 0.06, NFI = 0.92, IFI = 0.92, and CFI = 0.93) (Table 3). In the final model, the mediating effect explained 76% of variance variation in the dependent variable, and subjective sleep quality and sleep efficiency explained 53.6% and 22.4% of variance, respectively. In order to reduce the interaction between the mediating variables, the residual correlation analysis between the two mediating variables was conducted (path coefficient = 0.55).

The paths from the mediation model were examined using direct and indirect effects of the study variables (Table 4; Figure 2). The results showed the PA-induced indirect effect on inhibitory control performance while adjusting for sleep parameters among the university students. Thus, sleep parameters (subjective sleep quality and sleep efficiency) played a mediating role in the indirect relationships. Additionally, the nonsignificant direct effect of physical activity on inhibitory control performance is 0.001.

## 4. Discussion

Based on previous literature, the mediating role of sleep parameters on the relationship between PA and EF (i.e., inhibitory control) is expected, but this test has not been formally assessed in a young sample and with a wider range of sleep health indicators [12,32]. In the present study, we observed in our cohort of healthy younger adults (1) a negative correlation between PA and inhibitory control performance, (2) a negative correlation between PA and subjective sleep quality as well as sleep efficiency ((number of sleep hours/number of hours spent in bed) × 100), and (3) positive correlations between inhibitory control and sleep parameters (subjective sleep quality and sleep efficiency). Moreover, the mediation analysis reveals that subjective sleep quality and sleep efficiency play mediating roles in the PA–EF (inhibitory control).

In this study, positive correlations were observed between PA level and sleep indicators (subjective sleep quality and habitual sleep efficiency), which is in line with the findings of existing studies [26,33]. Over the last decades, it has been shown that, among younger adults, a higher PA level is positively associated with sleep health [34,35]. Notably, recent studies proposed that the PA–sleep relationship may be influenced by PA slots [36]. To be specific, some researchers emphasized that light PA in the daytime was correlated with better sleep status, whereas PA at night may reduce the sleep duration and sleep time window [37]. However, as we used the IPAQ to assess PA, the time of day in PA engagement was not used in the present study but such an assessment should be considered in future studies to deepen our understanding of the relationship between PA, sleep, and cognitive performance.

The results from our partial correlation analysis indicate that EF (inhibitory control) was positively correlated with both subjective sleep quality and sleep efficiency but not with sleep duration. Such results are consistent with previous researches in which sleep parameters were measured with an actigraphic device and polysomnography [38,39,40]. PA–sleep relationships may be partially explained by the following neurobiological mechanisms: (1) higher sleep efficiency and sleep quality enhanced the efficacy of slow-wave sleep in the restoration of prefrontal cortex function (which is responsible for inhibitory control performance) [41] and (2) prior sleep deprivation, sleep disorders, sleep fragmentation, and medication effects usually occur alongside longer sleep durations, which does not reflect real sleep health [42,43,44]. Non-restorative sleep caused by recurrent awakenings and stage shifts can even be observed in the population with normal sleep duration [45]. Due to this, sleep duration cannot be regarded as a reliable indicator for sleep health.

Only an indirect relationship (mediated by sleep parameters) rather than a direct relationship between PA and cognitive performance was observed among university students in the present study. This finding is partially supported Wilckens et al., who found that PA level was indirectly correlated with inhibitory control in both adolescent (aged 21–30) and older adults (aged 55–80) and that sleep efficiency but not total sleep time or sleep duration played a mediating role. In the present study, we observed that both sleep efficiency and subjective sleep quality played important roles in the PA–EF relationship. Based on these outcomes, it could be inferred that ease of falling asleep and staying asleep rather than any aspect of sleep affect the PA–cognition connections. Moreover, it is essential to highlight that the direct relationship between PA and inhibitory control performance reflects the overall relationship with cognition, which consists of sleep parameters and other mediators not tested here. Additionally, some of the potential mediators may suppress the association between PA and inhibitory control [46,47].

Besides the primary outcome carried out from the present study, some novel findings were summarized based on a comparison with previous research [28,29,48]. It is suggested that the mediating role of sleep efficiency in the PA–EF (inhibitory control) relationship is observed across different age groups [26]. PA can even attenuate the negative impact of low sleep efficiency on executive function, with the clearest effects observed using direct measurements of sleep and PA [49]. Even though sleep quality and sleep duration were also found to be positively associated with PA and cognition among young adults [33], an apparent functional weakening in such relationships appear in the aging population [27]. It seems that sleep measures such as sleep quality and sleep duration are more sensitive to aging-related neurobiology (e.g., neural atrophy, nocturnal hypoxia, neuroendocrine changes, and altered neuromodulation), which may reduce the potential to impact cognitive performance through strategies such as PA [50].

Finally, this study has a certain theoretical and practical significance, but some limitations must be acknowledged when interpreting our findings. First, the cross-sectional design does not allow us to make strong conclusions regarding the causality of the observed correlations. Secondly, this study did not account for the time of day when participants engaged in PA, which is known to influence sleep patterns and thus might influence cognitive performance, too. Thirdly, our sleep pattern assessment relies on the PSQI, which is an international recognized questionnaire with good psychometric properties but relies on subjective ratings rather than objective ratings. Given difference in subjective and objective ratings of sleep [48,51], this might have influenced our analysis. Thus, caution should be taken when comparing our results with findings of other studies quantifying sleep parameters by objective tools (e.g., actigraphy) although those studies point in the same direction (better sleep patterns are associated with better cognitive performance) [52]. In this regard, we recommend that further studies investigating the relationship between regular PA level, sleep patterns, and cognitive performance should consider assessing both subjective ratings and objective ratings of sleep. For studies with more stern requirements, polysomnography, the gold standard for sleep research, is recommended. Lastly, we focused only on one cognitive parameter (inhibitory control), preventing the generalizability of our results to other EF components and cognitive measures.

## 5. Conclusions

Sleep efficiency and subjective sleep quality were found to statistically mediate the significant relationship between physical activity and inhibitory control in a population of university students. Joint measurements including both objective and subjective assessment and participants in a wider age range should be considered in further studies to test the potential neurobehavioral mechanisms and moderators.

## Figures and Tables

**Table 1:** Demographic information on the variables.

	Males (*n* = 71)	Females (*n* = 109)	Total (*n* = 180)
Demographic and Anthropometric Parameters			
Age (years)	20.31 ± 1.45	19.68 ± 1.64	19.93 ± 1.59
BMI (kg/m^2^)	21.66 ± 2.05	21.20 ± 2.55	21.37 ± 2.37
International Physical Activity Questionnaire			
MET-min/w	4967.05 ± 1447.06	5171.22 ± 1556.39	5090.69 ± 1513.41
Pittsburgh Sleep Quality Index			
Subjective sleep quality	0.68 ± 0.69	0.76 ± 0.79	0.73 ± 0.75
Sleep time	0.62 ± 0.70	0.54 ± 0.69	0.57 ± 0.69
Sleep efficiency	0.73 ± 0.72	0.68 ± 0.76	0.70 ± 0.74
Sleep disorders	0.80 ± 0.71	0.97 ± 0.55	0.91 ± 0.62
Stroop Test			
Congruent accuracy (%)	95.00 ± 9.35	97.00 ± 5.80	96.00 ± 7.46
Incongruent accuracy (%)	95.00 ± 8.65	97.00 ± 4.70	96.00 ± 6.66
Congruent reaction time (ms)	899.98 ± 107.20	867.53 ± 135.39	875.47 ± 141.42
Incongruent reaction time (ms)	1000.18 ± 107.79	964.34 ± 148.10	973.07 ± 152.56
Stroop effect (ms)	100.21 ± 45.64	96.81 ± 47.13	98.15 ± 46.45

Note. BMI: body mass index; MET: metabolic equivalent of task.

**Table 2:** Correlation analyses among the outcome measures.

	MET-min/w	S1	S2	S3	S4	S5	S6	S7	Acc.1	Acc.2	RT1	RT2	Stroop Effect
**MET**	-												
**S1**	−0.425 ***	-											
**S2**	−0.033	0.068	-										
**S3**	−0.006	0.082	0.107	-									
**S4**	−0.519 ***	0.652 ***	0.033	0.126	-								
**S5**	0.180 **	0.100	0.186 **	−0.106	−0.008	-							
**S6**	0.153 **	0.060	0.125	−0.210 **	0.081	0.331 ***	-						
**S7**	0.058	0.047	0.289 ***	−0.039	0.002	0.198 **	0.412 ***	-					
**Acc.1**	−0.018	0.057	0.064	0.074	−0.023	0.103	−0.019	0.136	-				
**Acc.2**	−0.056	−0.032	−0.016	0.116	−0.062	−0.002	−0.106	0.017	0.486 ***	-			
**RT1**	0.036	0.139	0.021	−0.053	0.043	0.047	0.116	0.033	−0.076	−0.107	-		
**RT2**	−0.101	0.419 ***	0.036	−0.006	0.252 ***	0.060	0.142	0.032	−0.027	−0.109	0.938 ***	-	
**Stroop effect**	−0.386 ***	0.831 ***	0.045	0.125	0.611 ***	0.045	0.098	0.003	0.125	−0.026	0.008	0.355 ***	-

Note. MET = metabolic equivalent of task; S1 = subjective sleep quality; S2 = sleep latency; S3 = sleep duration; S4 = habitual sleep efficiency; S5 = sleep disturbances; S6 = use of sleep medication; S7 = daytime dysfunction. Acc.1 = congruent accuracy; Acc.2 = incongruent accuracy; RT1 = congruent reaction time; RT2 = incongruent reaction time. ** means that *p* value is less than 0.01; *** means that *p* value is less than 0.001.

**Table 3:** Modification indices in multiple mediation model 2.

Model	χ^2^	*p*	*f*	χ^2^/DF	NFI	IFI	CFI	RMSEA	SRMR
Initial model	64.43	0.001	1	64.43	0.83	0.83	0.82	0.07	0.06
Final model	0.06	0.06	1	0.06	0.92	0.92	0.93	0.05	0.04
Δχ^2^	64.37								

**Table 4:** The bootstrap analysis of multiple mediation model 2.

Route	Indirect Effects of Standardization	Average Indirect Effect	95% Confidence Interval		*p*
			Lower Limit	Upper Limit	
Subjective Sleep Quality	0.42 × 0.77 = 0.32	0.41	0.27	0.55	0.01
Sleep Efficiency	0.53 × 0.13 = 0.07	0.51	0.39	0.63	0.01

## Data Availability

The data presented in this study are available on request from the corresponding author.
